# Supplementary material for: Using a Very Low Energy Diet to Achieve Substantial Preconception Weight Loss in Women with Obesity: A Review of the Safety and Efficacy
Source: Nutrients. 2022 Oct 21;14(20):4423. doi: 10.3390/nu14204423 (PMC9608905; doi:10.3390/nu14204423)
Supplement: Supplementary file 1 [file nutrients-14-04423-s001.zip › nutrients-1946421-supplementary.pdf]

Table S1. Search terms used in systematic review.

Concept	Search Terms
Obesity	Obesity[tw] OR overweight[tw] OR weight[tw] OR "weight loss" [tw] OR "body weight" [tw] OR "Obesity"[Mesh]
Very Low Energy Diet	"Very Low Energy Diet" [tw] OR "Very Low Calorie Diet" [tw] OR VLED[tw] OR VLCD[tw] OR "Low Calorie Diet" [tw] OR "Ketogenic Diet" [tw] OR "Diet, Carbohydrate-Restricted"[Mesh]
Preconception	Preconcep*[tw] OR prepreg*[tw] OR pregnan*[tw] OR concep*[tw] OR "Preconception Care"[Mesh]
